# The YAP1–NMU Axis Is Associated with Pancreatic Cancer Progression and Poor Outcome: Identification of a Novel Diagnostic Biomarker and Therapeutic Target

**DOI:** 10.3390/cancers11101477

**Published:** 2019-09-30

**Authors:** Wonbeak Yoo, Jaemin Lee, Eunsung Jun, Kyung Hee Noh, Sangmin Lee, Dana Jung, Kwang Hwa Jung, Ji-Su Kim, Yun-Yong Park, Song Cheol Kim, Seokho Kim

**Affiliations:** 1 Environmental Disease Research Center, Korea Research Institute of Bioscience and Biotechnology, Daejeon 34141, Korea; yoow@kribb.re.kr; 2 Industrial Bio-Materials Research Center, Korea Research Institute of Bioscience and Biotechnology, Daejeon 34141, Korea; groove9495@gmail.com (J.L.); lsm9089@gmail.com (S.L.); eksk95@gmail.com (D.J.); 3 Department of Convergence Medicine, University of Ulsan College of Medicine, Asan Institute for Life Sciences, Asan Medical Center, Seoul 05505, Korea; eunsungjun@amc.seoul.kr; 4 Korea Research Institute of Bioscience and Biotechnology, Daejeon 34141, Korea; trollius@kribb.re.kr; 5 Department of Molecular and Human Genetics, Baylor College of Medicine, Houston, TX 77030, USA; kwanghwa.jung@bcm.edu; 6 National Primate Resources Center, Korea Research Institute of Bioscience and Biotechnology, Jeonbuk 56212, Korea; kimjs@kribb.re.kr; 7 Division of Hepato-Biliary and Pancreatic Surgery, Department of Surgery, Asan Medical Center, AMIST, University of Ulsan College of Medicine, Songpa-gu, Seoul 05505, Korea; 8 Department of Medicinal Biotechnology, College of Health Sciences, Dong-A University, Busan 49315, Korea

**Keywords:** pancreatic cancer, YAP1–NMU axis, metastasis, poor outcome

## Abstract

Yes-associated protein (YAP)-1 is highly upregulated in pancreatic cancer and associated with tumor progression. However, little is known about the role of YAP1 and related genes in pancreatic cancer. Here, we identified target genes regulated by YAP1 and explored their role in pancreatic cancer progression and the related clinical implications. Analysis of different pancreatic cancer databases showed that Neuromedin U (NMU) expression was positively correlated with YAP1 expression in the tumor group. The Cancer Genome Atlas data indicated that high YAP1 and NMU expression levels were associated with poor mean and overall survival. YAP1 overexpression induced NMU expression and transcription and promoted cell motility in vitro and tumor metastasis in vivo via upregulation of epithelial–mesenchymal transition (EMT), whereas specific inhibition of NMU in cells stably expressing YAP1 had the opposite effect in vitro and in vivo. To define this functional association, we identified a transcriptional enhanced associate domain (TEAD) binding site in the NMU promoter and demonstrated that YAP1–TEAD binding upstream of the NMU gene regulated its transcription. These results indicate that the identified positive correlation between YAP1 and NMU is a potential novel drug target and biomarker in metastatic pancreatic cancer.

## 1. Introduction

Pancreatic cancer, which is the fourth most common cause of cancer-related mortality in the US, has the lowest five-year survival rate (8%) among solid tumors. Pancreatic cancer is a highly invasive and metastatic, and the clinical outcome of patients is poor [1,2]. Identifying preventive or therapeutic strategies for pancreatic cancer is critical to improve its early detection and prevent its progression.

The Yes-associated protein (YAP, also known as YAP1 or YAP65) YAP1 and its interacting family of transcriptional co-factors known as transcriptional enhanced associate domain (TEAD) are main components of the Hippo signaling pathway and are associated with cell growth, proliferation, migration, motility, and organ development [3,4,5]. YAP1 is an oncoprotein involved in tumorigenesis in multiple types of cancer including pancreatic cancer [6,7,8,9,10]. YAP1 activation promotes epithelial–mesenchymal transition (EMT) and is associated with liver metastasis in pancreatic cancer, thereby inducing progression to a more aggressive phenotype [11,12].

In the past decade, large-scale molecular analyses and gene expression studies identified several YAP1-related and downstream target genes [13,14,15]. Clinicopathological and biological analyses suggest that the expression of YAP1 target genes is correlated with tumor progression in non-small cell lung cancer [5,16,17], triple-negative breast cancer [18,19,20], and colorectal carcinoma [21,22]. Activation of YAP1 and its downstream target genes is associated with resistance to DNA-damaging agents, UV, and radiation in different types of cancer [18,23,24,25,26,27,28,29]. Moreover, studies on YAP1 and its target genes have focused on the identification of diagnostic factors and the development of anticancer drugs. Despite the central role of YAP1, its downstream target genes and related mechanisms of action remain poorly understood.

Neuromedin U (NMU), a secreted neuropeptide belonging to the neuromedin family, was originally isolated from porcine spinal cord and has been related to multiple physiological functions involved in stress, immune response, obesity, and energy metabolism [30,31,32,33]. However, a limited number of studies have reported on NMU association with cancer.

Here, we identified NMU as a critical factor associated with pancreatic cancer metastasis and poor prognosis through database screening and analysis of clinical samples. We identified an association between NMU and YAP1 and provide in vivo and in vitro evidence supporting the essential role of NMU in YAP1-driven pancreatic cancer progression and metastasis. These findings suggest that a novel mechanism involving YAP1 and NMU could play a role in pancreatic cancer progression and could serve as a clinical therapeutic target for new therapies for pancreatic cancer patients.

## 2. Results

### 2.1. Identification of YAP1 Target Genes in Human Pancreatic Cancer

**Figure 1:**
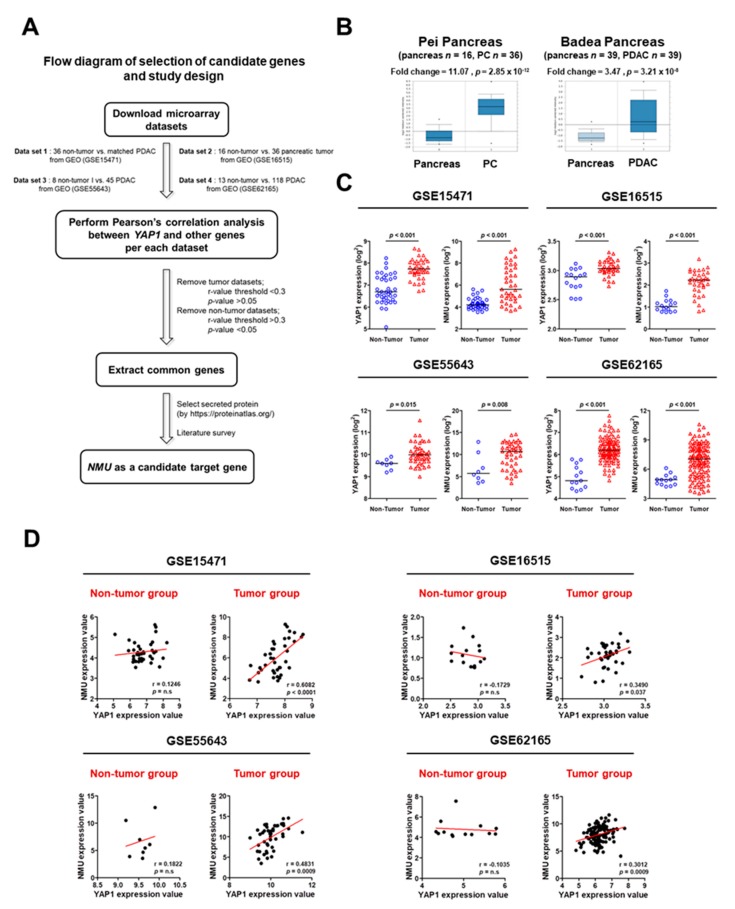
Construction of prediction models and validation in multiple databases. (**A**) Schematic overview of target gene identification. (**B**) Validation of Neuromedin U (NMU) expression in the cohort of Pei and Badea from Oncomine. (**C**) Validation of Yes-associated protein 1 (YAP1) and NMU expression in cohorts from the GEO dataset. (**D**) Pearson’s correlation analysis of YAP1 and NMU in cohorts from the GEO dataset.

Potential YAP1 target genes were identified by searching four independent GEO dataset profiles. Gene expression in tumor and non-tumor tissues was statistically analyzed using the Student’s *t*-test, and Pearson’s correlation was used to analyze each of the four datasets. In the correlation analysis, top-ranked common overlapping genes with significant *p*-values were selected from the tumor group only. These genes were then used to identify secreted candidates in the protein atlas (https://www.proteinatlas.org). NMU consistently showed high levels of correlation with YAP1 in multiple datasets and was thus identified as a candidate YAP1 interactor (Figure S1). The workflow of the study is shown in Figure 1A. NMU mRNA expression in pancreatic cancer and pancreatic ductal adenocarcinoma (PDAC) tissues was validated by Oncomine data-mining analysis. The mRNA level of NMU was significantly increased in the Pei pancreas dataset (fold change = 11.07; *p* = 2.85 × 10^−12^) and the Badea pancreas dataset (fold change = 3.47; *p* = 3.21 × 10^−8^) (Figure 1B). Differences in the mRNA expression of NMU and YAP1 between tumor and non-tumor tissues were identified using four GEO databases, which showed that NMU and YAP1 mRNA expression levels were significantly higher in tumor than in non-tumor tissues (Figure 1C). Next, the four datasets of pancreatic cancer were analyzed using Pearson’s correlation analysis to determine the relationship between NMU and YAP1 mRNA expression. As shown in Figure 1D, only the tumor group showed a significant positive correlation between NMU and YAP1 (GSE15471 *r* = 0.6082, *p* < 0.0001; GSE16515 *r* = 0.3490, *p* = 0.037; GSE55643 *r* = 0.4831, *p* = 0.0009; GSE62165 *r* = 0.3012 *p* = 0.0009). Collectively, these data suggest that NMU expression is positively regulated by YAP1 or that they are involved in the same pathway.

### 2.2. High NMU and YAP1 Expression Levels Correlate with Poor Prognosis in Human Pancreatic Cancer Patients

**Figure 2:**
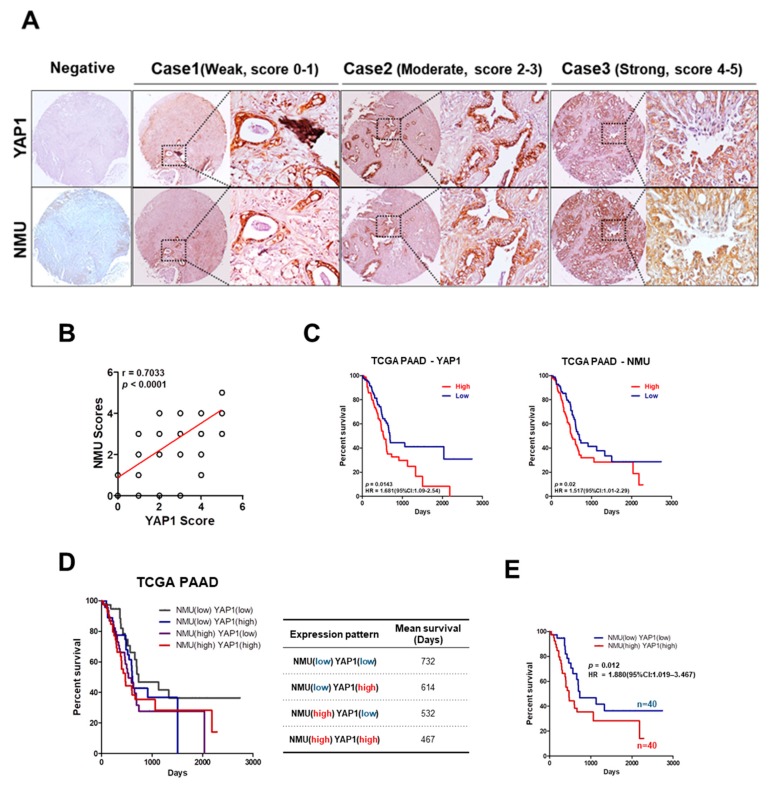
Clinical significance of the correlation between YAP1 and NMU expression for predicting the prognosis of pancreatic cancer. (**A**) A tissue microarray (TMA) was used to perform immunohistochemical (IHC) staining of human pancreatic cancer samples. Representative IHC images from the TMA. Scores indicated weak, moderate, and strong positive staining in tumors. Magnification: 40×. (**B**) Correlation between YAP1 and NMU expression in the TMA. Pearson’s correlation coefficient was used to quantify the correlation between the expression of YAP1 and that of NMU in tumors. (**C**) Kaplan–Meier survival analysis of patients from The Cancer Genome Atlas (TCGA) according to YAP1 and NMU expression levels; *p*-values derived from the log-rank test are indicated in each comparison. (**D**,**E**) Kaplan–Meier survival analysis and mean survival rates of patients according to YAP1 and NMU expression levels. Comparison of patients from TCGA expressing YAP1 and NMU at high or low levels; *p*-values derived from the log-rank test are indicated in each comparison. PAAD: Pancreas adenocarcinoma.

A tissue microarray (TMA) containing 66 pancreatic cancer samples showed marked variation in YAP1 and NMU expression. Therefore, YAP1 and NMU expression was categorized into three groups according to the staining intensity, i.e., weak (score 0–1), moderate (score 2–3), and strong (score 4–5) staining intensity (Figure 2A). A strong correlation between YAP1 and NMU expression was observed, confirming the results of Figure 1 (Figure 2B). To determine the prognostic value of YAP1 and NMU expression in patients with pancreatic cancer, we compared the outcomes of patients with low expression (*n* = 87) and high expression (*n* = 87) of these proteins. As shown in Figure 2C, the group with high YAP1 or NMU expression had significantly poorer survival than the low-expression group. Analysis of the combined effect of NMU and YAP1 on clinical outcomes showed that high NMU and YAP1 expression was associated with poor mean survival in pancreatic cancer patients. The mean survival was 467 days in the high-NMU/YAP1-expression group and 732 days in the low-NMU/YAP1-expression group (Figure 2D). Patients with high NMU and high YAP1 expression had the poorest overall survival [*p* = 0.012, HR = 1.880, 95% CI: 1.019–3.467] (Figure 2E). These results strongly support an association between YAP1/NMU expression and poor outcome in pancreatic cancer.

### 2.3. YAP1 Enhances NMU Expression in Pancreatic Cancer Cells

**Figure 3:**
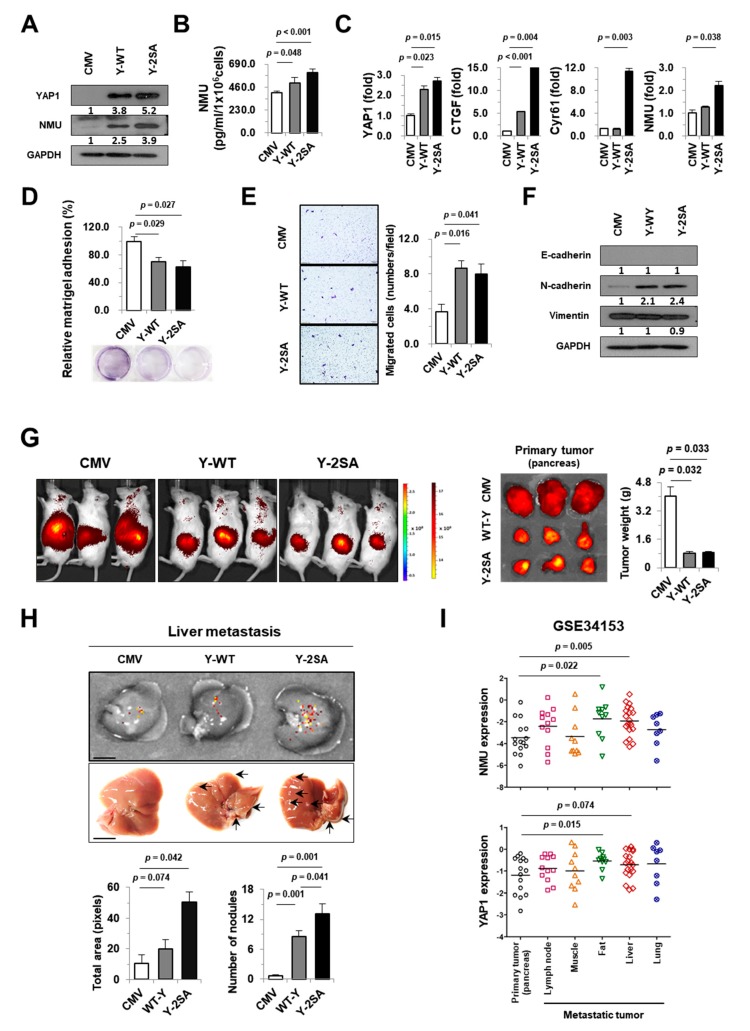
YAP1 overexpression promotes NMU expression and correlates with liver metastasis in vitro and in vivo. (**A**) Western blot analysis of YAP1 and NMU in the indicated cells. Y-WT: cells overexpressing wild-type (WT) YAP1), Y-2SA: cell overexpressing constitutively active YAP S217/381A. (**B**) Concentration of secreted NMU in the culture medium from MIA PaCa-2 cells. (**C**) Changes in YAP1, YAP1-target genes, and NMU mRNA expression assessed by real-time qPCR. (**D**) Cell adhesion analysis. Adherent cells were stained and counted, and the proportion of stained cells was expressed as a percentage with respect to the control. (**E**) Migration ability assessed by the Transwell assay as described in Materials and Methods. Representative images of migrating cells are displayed (left) with quantification (right) (magnification, ×100). (**F**) Western blot analysis of epithelial–mesenchymal transition (EMT) markers with the indicated antibodies. (**G**) Representative bioluminescence images of primary pancreatic tumors. Gross and final weights were determined 6 weeks after orthotopic cancer cell injection (*n* = 5/group). (**H**) Bioluminescence images of metastatic livers and number of tumor nodules. Scale bar: 1 cm. (**I**) Validation of the metastatic properties of YAP1 and NMU expression in pancreatic primary and metastatic tumors from the GEO dataset GSE34153. Values are expressed as the mean ± SEM.

Next, we examined the effects of YAP1 on the regulation of NMU gene expression in tumor cells in vitro. The MIA PaCa-2 cell line, which has the lowest expression levels of YAP1 and NMU among the pancreatic cancer cell lines tested, was chosen as an adequate in vitro tumor model (Figure S2). YAP1 overexpression increased NMU protein, secretion, and the expression of YAP1 downstream target genes *CTGF* and *Cyr61* (Figure 3A–C). The effect of YAP1 overexpression on metastasis was examined using cell adhesion and migration assays. YAP1 overexpression decreased cell adhesion and increased cell migration (Figure 3D,E). The YAP1-overexpressing cells Y-WT (expressing wild-type (WT) YAP1) and Y-2SA (expressing constitutively active YAP S217/381A) showed downregulation of the epithelial marker E-cadherin and upregulation of the mesenchymal marker N-cadherin, with no differences in the expression of vimentin (Figure 3F). The metastatic properties of YAP1-overexpressing pancreatic cancer cells were examined in immune-deficient NSG mice orthotopically injected with pancreatic cancer cells. Tumor growth was monitored for 6 weeks by whole-body immunofluorescence imaging, after which mice were sacrificed, and the liver, lung, spleen, and kidney were excised and scanned for metastatic cells. At 6 weeks, tumors were significantly larger in mice injected with CMV control cells than in those injected with YAP1-overexpressing cells (Y-WT and Y-2SA) (Figure 3G). There were no significant differences in organ weight or in the incidence of metastasis between the different tissues (Figure S3). The incidence of liver metastasis in Y-2SA-injected mice was higher compared with those of the CMV and Y-WT groups (Figure 3H). To further examine the mechanism underlying the association between YAP1 and metastasis, we screened the human GEO database to identify differences in NMU and YAP1 mRNA expression between primary and metastatic tumors (Figure 3I). The results showed that NMU and YAP1 mRNA expression levels were higher in metastatic fat and liver tumors than in primary pancreatic tumors. Taken together, the results of the adhesion and migration assays, investigation of molecular changes, and xenograft mouse model suggest that YAP1 plays an important role in NMU expression and affects the metastatic properties of pancreatic cancer.

### 2.4. NMU Is Involved in YAP1-Induced Aggressiveness of Pancreatic Cancer

**Figure 4:**
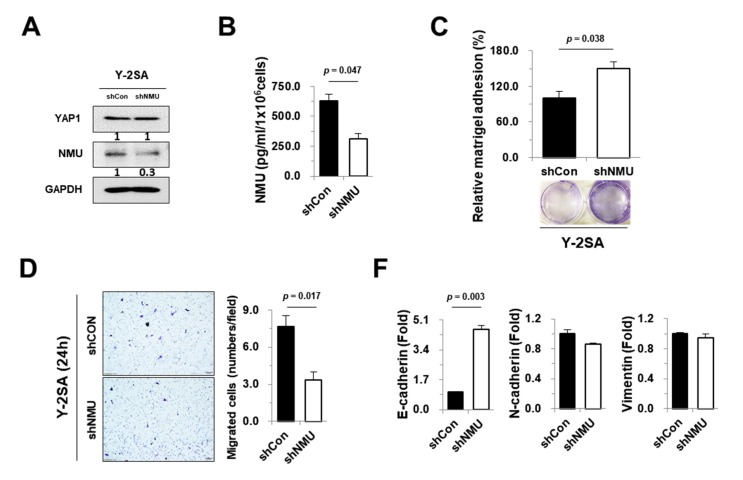

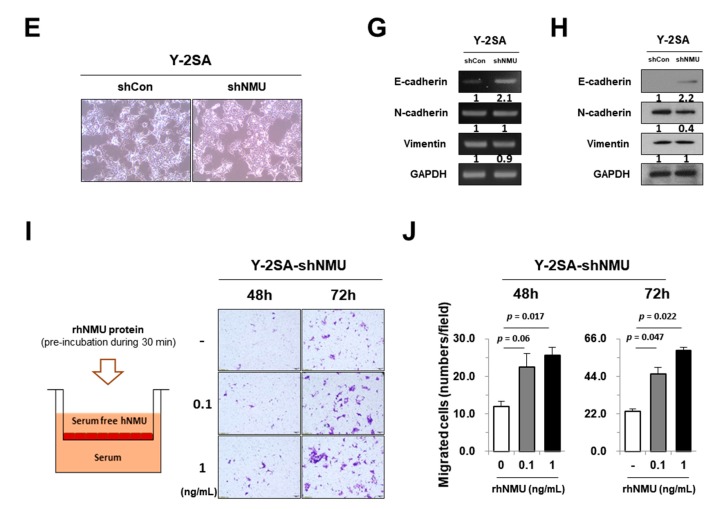
Effects of NMU downregulation on YAP1-overexpressing cells. (**A**) Western blot analysis of YAP1 and NMU in the indicated cells. (**B**) Concentration of secreted NMU in the culture medium. (**C**) Cell adhesion analysis. Adherent cells were stained and counted, and the proportion of stained cells was expressed as a percentage with respect to the control. (**D**) Migration ability assessed by the Transwell assay as described in Materials and Methods. Representative images of migrating cells are displayed (left) with quantification (right) (magnification, ×100). (**E**) Morphological changes of Y-2SA-overexpressing MIA PaCa-2 cells with or without shNMU (magnification, ×100). (**F**,**G**) Changes in mRNA expression of EMT markers assessed by real-time qPCR and RT-PCR. (**H**) Western blot analysis of EMT markers was performed with the indicated antibodies. (**I**) Flow diagram and migration ability assessed by the Transwell assay as described Materials and Methods (magnification, ×100). (**J**) Quantification of migrating cells in the lower chamber. Values are expressed as the mean ± SEM.

YAP1 upregulates NMU expression and promotes tumor metastasis. To confirm the association between YAP1-modulated NMU expression and tumor incidence and metastasis, we constructed stable Y-2SA cell lines transfected with shRNA against NMU (Y-2SA-shNMU). The knockdown efficiency of NMU in Y-2SA cells was confirmed by western blotting and ELISA (Figure 4A,B). No significant differences were found in organ weight or in the incidence of metastasis between the different tissues (Figure S4). Although cell proliferation was not affected by NMU knockdown in our data, shNMU cells had increased cell adhesion and diminished cell migration and epithelial cell morphology compared with Y-2SA cells (Figure 4C–E). NMU knockdown upregulated the gene and protein levels of E-cadherin and downregulated the protein levels of N-cadherin (Figure 4F–H). YAP1 overexpression or knockdown led to differences in the expression and secretion of NMU and was associated with YAP1–NMU-mediated cell motility. Human recombinant NMU (rhNMU) increased the migration of Y-2SA-shNMU cells in a dose- and time-dependent manner (Figure 4I,J). These results suggest that NMU may play a role in the modulation of the metastatic properties enhanced by YAP1.

**Figure 5:**
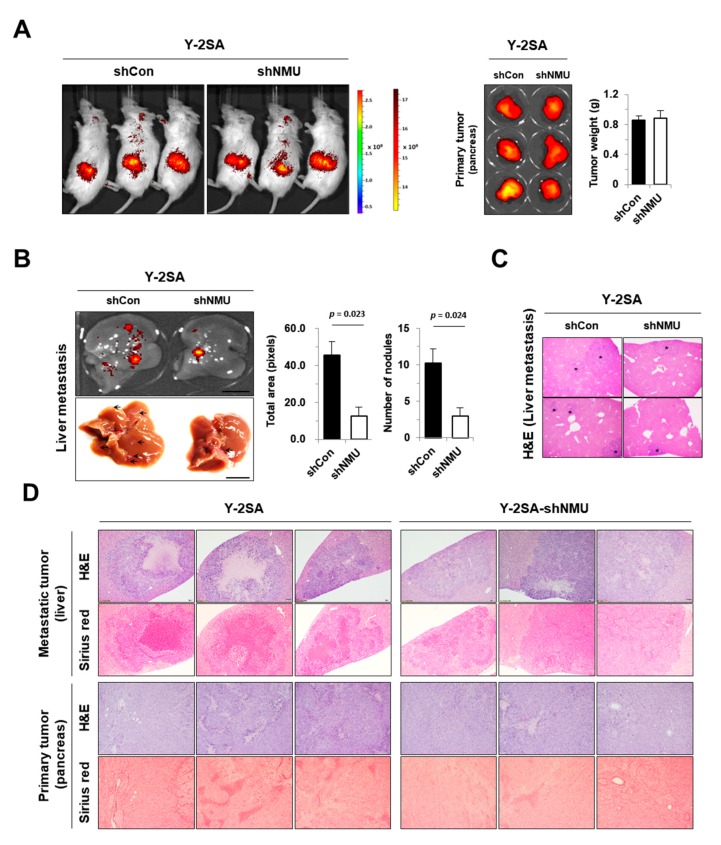
NMU is essential for liver metastasis induced by YAP1 in pancreatic cancer. (**A**) Representative bioluminescence images of primary pancreatic tumors. Gross and final weights were assessed at 6 weeks after orthotopic cancer cell injection (*n* = 5/group). (**B**) Bioluminescence images of metastatic livers and number of tumor nodules. Scale bar: 1 cm. (**C**) Hematoxylin and eosin (H & E) staining of sections of mouse liver nodules in the Y-2SA-shCON group compared with the Y-2SA-shNMU group (magnification, ×10). (**D**) Representative H & E and Sirius red staining images of metastatic and primary tumors (magnification, ×40). Values are expressed as the mean ± SEM.

The effect of NMU inhibition in vivo was examined in Y-2SA-shNMU cells because these cells showed decreased motility and recovered MET compared with Y-2SA cells. Mice were sacrificed at 6 weeks after tumor implantation. Although there were no significant differences in primary tumor bioluminescence and weight (Figure 5A), Y-2SA-shNMU cells-injected mice had fewer metastatic liver nodules than mice injected with Y-2SA-shCON cells (Figure 5B,C). Hematoxylin and eosin (H & E) staining was used to directly observe the metastatic lesions inside the liver. Collagen accumulation in metastatic and primary tumors was lower in the Y-2SA-shNMU-injected group than in the Y-2SA-shCON-injected group (Figure 5D). Collectively, these findings indicated that NMU downregulation was responsible for the suppressive effects of YAP1-driven cell motility and metastatic properties and confirmed the results obtained by the overexpression of YAP1.

### 2.5. NMU Is a Direct Target of YAP1/TEAD

**Figure 6:**
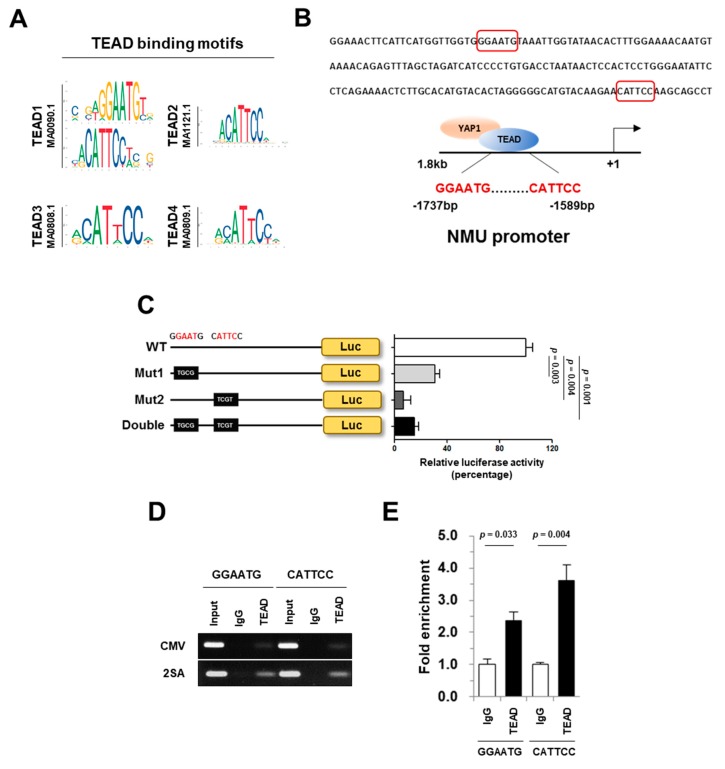
NMU is transcriptionally activated by transcriptional enhanced associate domain (TEAD). (**A**,**B**) The binding motif of TEADs and the sequences were obtained from JASPAR. (**C**) Diagram of plasmids encoding the wild-type (WT) or mutant (Mut1, Mut2, and Double: Mut1 and Mut2) NMU promoter cloned into the pGL2 vector containing the luciferase gene. (**D**) Strategy used for ChIP assay using the putative DNA-binding region of the NMU promoter amplified by PCR. Input was included as a positive control, and IgG was used as a negative control. (**E**) ChIP assay followed by qPCR to quantify the binding of TEADs to the proximal promoter regions of the indicated genes. Values are expressed as the mean ± SEM.

YAP1 is a transcriptional co-activator that interacts with TEAD DNA binding proteins to initiate proliferative and oncogenic activities [14,16]. Our results led us to hypothesize that NMU expression may be positively regulated by YAP1. To confirm this hypothesis, TEAD binding motifs were predicted using the JASPAR database (Figure 6A), and the analysis of the human NMU promoter region identified two potential TEAD binding elements located between base pairs −1737 and −1732 (5′-GGAATG-3′) and −1589 and −1584 (5′-CATTCC-3′) upstream of the transcription start site (+1). The cells 293FT were transfected with a NMU promoter–luciferase reporter constructs with (WT-NMU promoter, WT) or without (Mutant1-NMU promoter, Mut1; Mutant2-NMU promoter, Mut2; double-mutant-NMU promoter, Double) the putative TEAD response element (Figure 6B). The results of the reporter assay showed that mutation of the TEAD binding elements in the NMU promoter greatly decreased NMU transcriptional activity in 293FT cells (Figure 6C). To determine whether NMU is a direct transcriptional target of YAP1/TEAD, we performed a ChIP assay in 293FT cells, which confirmed the binding of TEAD to the promoter region of NMU (Figure 6D). Taken together, these results indicated that TEAD binds directly upstream of the NMU gene to regulate its transcription.

## 3. Discussion

Recent advances in microarray and sequencing technologies have led to the development of new strategies for cancer diagnosis, treatment, and prognosis prediction [20,34,35]. These strategies can also be used to define cancer subtypes, predict cancer metastasis, and determine the clinical outcomes of patients [36,37,38,39]. YAP1 is frequently upregulated and activated in different cancers including pancreatic cancer and contributes to tumor initiation and progression [16]; however, there are few studies on YAP1 and its downstream targets and mechanisms. Here, we established an effective screening system to identify YAP1-related genes using multiple independent pancreatic cancer cohorts. The strategy was as follows: (i) identification of positively correlated genes in four independent GEO datasets through genome-wide gene expression profiling; (ii) validation of gene expression analysis using the Oncomine and GEO databases; (iii) confirmation of the clinicopathological significance of candidate genes using tissue microarray and clinical outcomes; and (iv) verification of the roles of YAP1 and candidate genes in tumor progression and metastasis in vitro and in vivo. This screening system identified 25 genes correlated with YAP1 expression in the cancer group, among which we selected NMU as the most significantly overexpressed gene in patient samples and cancer cell lines. We showed that the YAP1–NMU correlation promoted cancer metastasis and was associated with poor prognosis in databases and clinical samples (Figure 1, Figure 2 and Figure 3I).

NMU is a secreted neuropeptide that is highly conserved in mammals [40]. This peptide is involved in different physiological functions, including smooth muscle contraction, energy homeostasis, appetite, blood pressure, body temperature, and hormone release [41,42,43,44]. A few studies investigated the association of NMU with cancer; however, the role of NMU in cancer remains unclear. In pancreatic cancer, Ketterer et al. reported that NMU is involved in cell migration, invasion, and dissemination via HGF–c-Met signaling [45]. Lee et al. identified NMU as a candidate biomarker for pancreatic intraepithelial neoplasia [46]. The present results suggest that the novel YAP1–NMU axis might be a diagnostic marker for predicting the progression and outcomes of patients with pancreatic cancer, as well as a therapeutic target.

We showed that YAP1–NMU signaling promoted EMT and collagen accumulation. Desmoplasia is characterized by excessive collagen deposition contributing to increased proliferation, migration, metastasis, EMT, and chemoresistance in pancreatic cancer cells. Indeed, the fibrous stroma is composed mainly of type I collagen [47,48,49,50]. Laklai et al. showed that YAP signaling impaired TGF-β activation in association with pancreatic cancer progression in vivo, leading to the accumulation of a fibrotic matrix associated with aggressive tumors and poorer overall survival [51]. This is consistent with the present results showing that YAP1–NMU crosstalk is responsible for collagen accumulation, aggressive tumor types, and metastatic properties in experimental models (Figure 5). Survival analysis showed that NMU/Col1a1/YAP1 expression was associated with poor overall survival, and NMU expression was the highest in pancreatic adenocarcinoma patients with pancreatitis in TCGA (Figure S5). Further study is needed to evaluate the role of the fibrotic process in tumor progression and metastasis in YAP1–NMU-expressing pancreatic cancer.

Luciferase reporter and ChIP assays based on the JASPAR database indicated that activated YAP1 modulates NMU expression (Figure 6). This is the first report showing that YAP1/TEAD directly regulate NMU, indicating that a YAP1/TEAD/NMU pathway may be important for YAP1-dependent tumor metastasis and the poor outcome of pancreatic cancer. A combined inhibition of YAP1, which is one of the major oncogenic drivers, and its downstream targets such as NMU may be effective to prevent cancer progression and metastasis in pancreatic cancer.

## 4. Materials and Methods

### 4.1. Cell Culture

The human pancreatic cancer cell lines BxPC-3, Capan-1, CFPAC-1, MIA PaCa-2, and PANC-1 were purchased from American Type Culture Collection. BxPC-3 cells were cultured in RPMI-1640 medium (Hyclone, Rockford, IL, USA) supplemented with 10% fetal bovine serum (FBS; Hyclone) and 1% antibiotic–antimycotic solution (AA; Gibco, Grand Island, NY, USA). MIA PaCa-2 and PANC-1 cells were maintained in Dulbecco’s modified Eagle’s medium (DMEM; Hyclone) supplemented with 10% FBS and 1% AA. CFPAC-1 cells were grown in Iscove’s modified Dulbecco’s medium (IMDM; Hyclone) supplemented with 10% FBS and 1% AA, and Capan-1 cells were cultured in IMDM with 20% FBS and 1% AA. All cell lines were maintained at 37 °C in a humidified atmosphere with 5% CO_2_.

### 4.2. Production of YAP1-Expressing Lentivirus

For YAP overexpression, the CMV, wild-type YAP1 (Y-WT), and YAP1-2SA (Y-2SA, constitutively active YAP S217/381A) vectors (Red fluorescent protein, RFP-tagged) were kindly donated by Dr. Dae-Sik Lim, Korea Advanced Institute of Science and Technology, Korea. Briefly, lentiviruses were generated by co-transfecting 293FT cells with packaging and envelope plasmids (psPAX2 and pMD2G) using Lipofectamine 3000 (Life technologies, Grand Island, NY, USA). Virus-containing supernatants were collected on days 1 and 2 after transfection and transferred into the MIA PaCa-2 cell line. For stable cell lines, RFP-positive cells were sorted using a FACS Aria IIu instrument (Becton Dickinson, San Jose, CA, USA), and selected cells were prepared for further experiments.

### 4.3. Mouse Experiment and Bioluminescent Imaging

NOD-scid gamma (NSG) mice were purchased from the Jackson Laboratory and maintained in a specific pathogen-free animal facility (KRIBB, Daejeon, Korea). Mouse models of pancreatic cancer were established in nude mice by orthotopic transplantation of 5 × 10^6^ MIA PaCa-2 cells with plasmids [MIA PaCa-2-CMV (CMV), MIA PaCa-2-WT YAP1 (Y-WT), MIA PaCa-2-YAP1 2SA (Y-2SA), and MIA PaCa-2-YAP1 2SA-shNMU (shNMU)] stably expressing fluorescence, which were monitored by weekly bioluminescent imaging. For bioluminescent imaging, mice were anesthetized with isoflurane and imaged using a Xenogen Spectrum in vivo imaging system (IVIS Lumina II; Caliper Life Sciences). All animal studies were performed in accordance with experimental protocols approved by the animal ethics committees of KRIBB (KRIBB-AEC-18162).

### 4.4. Measurement of Secreted Protein

The concentration of human NMU in cell culture supernatants was measured using a competitive ELISA kit (S-1253.0001, Peninsula Laboratories, San Carlos, CA, USA) according to the manufacturer’s protocol.

### 4.5. Cell–Matrix Adhesion and Transwell Migration Assays

For cell–matrix adhesion assays, 5 × 10^5^ cells were seeded in each well of a Matrigel-coated six-well plate. After incubation for 1 h, unbound cells were removed, and adherent cells were fixed with 4% paraformaldehyde (PFA) and stained with 0.4% crystal blue. Cells were washed with PBS and imaged using an Olympus microscope (Olympus, Tokyo, Japan) with a low-power objective. Complete medium was added to the lower chambers, and 5 × 10^4^ cells were placed in the upper chambers of 24-well Transwell plates (BD Biosciences, San Jose, CA, USA) in serum-free medium. After 24 h, non-migratory cells on the upper side of the inserts were removed. Migratory cells attached to the lower side of the inserts were fixed with 4% PFA and stained with 0.4% crystal blue. Cells were washed with PBS and imaged using an Olympus microscope. Stained cells were counted under a light microscope in three random fields of vision. The migration experiments were performed in duplicates, and the average number of migrated cells was determined from three independent experiments.

### 4.6. RNA Interference

Stable NMU knockdown was achieved using a lentivirus-mediated shRNA system. Human lentiviral pGFP-shNMU vectors (#TL311154) were purchased from OriGene Technologies, Inc. (Rockville, MD, USA). For packaging lentivirus, the resulting plasmids were co-transfected into HEK293FT cells with psPAX2 and pMD2G plasmids. The lentiviral supernatant was collected after 24 and 48 h of transfection and immediately used to infect MIA PaCa-2-YAP1 2SA cells for generating stable cell lines overexpressing shNMU and shCON (pLenti-GFP). After 12 h, the medium was replaced with normal growth medium. For stable cell lines, GFP-positive cells were sorted and prepared as described previously.

### 4.7. Western Blotting

Whole cell lysates were prepared using Pro-PREP Protein Extraction Solution (iNtRON Biotechnology, Seoul, Korea). Western blot analysis was performed as previously described [52] with the following antibodies: GAPDH, YAP1 (Cell Signaling, Danvers, MA, USA), E-cadherin, N-cadherin, vimentin (Santa Cruz Biotechnology, Santa Cruz, CA, USA), and NMU (Alpha Diagnostic International, TX, USA). Original western blots data were shown in Figure S6.

### 4.8. Reporter Constructs and Luciferase Activity

The YAP/TEAD binding elements were predicted using the JASPAR database (http://jaspar.genereg.net/). An NMU promoter including the YAP/TEAD binding site was obtained with NMU upstream regions generated by PCR using primers including the KpnI recognition site 5′-CCGGTACCCACCAGACGGACAAAGG-3′ and XhoI recognition site 5′-CCCTCGAGCACCTC TGTGGAAGCAC-3′, and ligated into the pGL2 vector (Promega, Madison, USA) directly. For mutagenesis of the NMU promoter, site-directed mutagenesis was performed following standard protocols as follows: mutation 1, 5′-CATTCATGGTTGGTGGGAATGTAAATTGGTATAAC-3′ to 5′-CATTCATGGTTGGTGGTGGCGTAAATTGGTATAAC-3′, and mutation 2, 5′-ATGTACAAGAACATTCCAAGCAGCCTGGTTCATG-3′ to 5′-ATGTACAAGAACTCGTCAAGCAGCCTGGTTCATG-3′. Successful incorporation of the mutations was confirmed by DNA sequencing. For luciferase assays, 293FT cells were seeded in 24-well plates, and reporter constructs with or without the YAP1 construct and Renilla were co-transfected (Figure 6C). After 24 h, the medium was removed, and the cells were harvested for luciferase assays (Dual Luciferase Assay kit, Promega). Luciferase activity was normalized to Renilla luciferase activity.

### 4.9. Chromatin Immunoprecipitation (ChIP) Analysis

For ChIP assays, cells were fixed, collected, and lysed according to the manufacturer’s protocols (Abcam, Cambridge, MA, USA). Protein–DNA complexes were immunoprecipitated overnight at 4 °C with an anti-TEAD antibody (#13295, Cell Signaling) or control IgG. The DNA extracted from each precipitate was resuspended and amplified by PCR using the following flanking primers: A, 5′-GAAGTCACACTCACCAGA-3′ and 5′-GTTATTAGGTCACAGGGG-3′; and B, 5′-CCCCTGTGACCTAATAAC-3′ and 5′-CAGCAGCTCAGCAAAATA-3′.

### 4.10. RNA Isolation and Gene Expression Analysis

Total RNA was extracted using the TRIzol reagent (Invitrogen, Eugene, OR, USA), according to the manufacturer’s protocol. cDNA was synthesized using an RT Kit (Biofact™, Daejeon, Korea). Quantitative real-time PCR was performed using Power SYBR green PCR master mix (Applied Biosystems, Foster City, CA, USA). Values were expressed as fold change with respect to the housekeeping gene GAPDH. Primer information is provided in Table S1.

### 4.11. Tissue Microarray and Reagents

A TMA for pancreatic cancer was purchased from US Biomax (PA721a, Rockville, MD, USA) and immunostained with anti-YAP1 (#12395, Cell Signaling) and anti-NMU (NMU41-A, Alpha Diagnostics International Inc., San Antonio, TX, USA) antibodies using a Dako REAL EnVision Detection System (Dako) following the manufacturer’s instructions. The cutoff value was defined on the basis of the method reported by Li et al. [53] with slight modifications, such as using the expression range 0–5 to divide samples according to low and high expression of YAP1 and NMU. All scores were evaluated separately; the mean calculated score was used to represent the immunoreactivity of the tumor. The correlation between YAP1 and NMU was evaluated by Pearson’s correlation, and the association between ordinal variables was assessed.

### 4.12. Bioinformatics Analyses

The pancreatic cancer patient datasets used in this study included GSE15471, GSE16515, GSE34153, GSE55643, GSE62165, and The Cancer Genome Atlas (TCGA). Correlations between YAP1 and the genes listed in Supplementary Figure S1 were evaluated by Pearson’s correlation using GraphPad Prism 5 (Version 5.0, GraphPad Software, Inc., San Diego, CA, USA). The expression pattern of NMU was determined by examining NMU mRNA levels in human cancers using datasets from the publicly available Oncomine database (http://www.oncomine.org). OncoLnc (http://www.oncolnc.org) and SurvExpress (http://bioinformatica.mty.itesm.mx/SurvExpress) were used to analyze the survival of PDAC patients with different protein expression levels, and the log-rank test was used to obtain a p-value for the significance of Kaplan–Meier curve divergence. Hazard ratios (HRs) and *p*-values were determined by Cox proportional hazards regression.

### 4.13. Statistical Analysis

Graphing and statistical analysis (the Student’s *t*-test or one-way analysis of variance for multiple comparisons) were performed using GraphPad Prism 5 (Version 5.0, GraphPad Software, Inc., San Diego, CA, USA). Data are presented as the mean ± standard error of the mean (SEM). Differences were considered statistically significant at *p* < 0.05.

## 5. Conclusions

In this study, we performed a correlation analysis of multiple independent databases to identify potential targets of YAP1 in pancreatic cancer. The results showed that NMU expression was highly correlated with YAP1 expression in pancreatic cancer. We showed that NMU is a direct transcriptional target of YAP1/TEAD and promotes cell motility and metastasis. These findings identify a novel molecular link between YAP1–NMU expression and pancreatic cancer metastasis and poor prognosis and suggest that targeting YAP1 and NMU could be a new treatment strategy for pancreatic cancer.

## Supplementary Materials

The following are available online at https://www.mdpi.com/2072-6694/11/10/1477/s1, Figure S1: Venn diagram of differentially expressed genes in the GSE15471, GSE16515, GSE55643, and GSE62165 datasets for the comparisons in Figure 1A, Figure S2: Expression levels of YAP1, its target genes, and NMU in human PDAC cells, Figure S3. Final body, liver, kidney, and spleen weights for CMV, Y-WT, and Y-2SA orthotopic tumor-bearing mice, Figure S4: Final body, liver, kidney, and spleen weight for Y-2SA-shCON and Y-2SA-shNMU orthotopic tumor-bearing mice, Figure S5: Kaplan–Meier survival analysis from TCGA, Figure S6: Original western blots data. Table S1. Primer information.
