# Supplementary material for: The YAP1–NMU Axis Is Associated with Pancreatic Cancer Progression and Poor Outcome: Identification of a Novel Diagnostic Biomarker and Therapeutic Target
Source: Cancers (Basel). 2019 Sep 30;11(10):1477. doi: 10.3390/cancers11101477 (PMC6826421; doi:10.3390/cancers11101477)
Supplement: Supplementary file 1 [file cancers-11-01477-s001.pdf]

Supplementary Materials: The YAP1-NMU Axis Is Associated with Pancreatic Cancer Progression and Poor Outcome: Identification of a Novel Diagnostic Biomarker and Therapeutic Target

Wonbeak Yoo, Jaemin Lee, Eunsung Jun, Kyung Hee Noh, Sangmin Lee, Dana Jung, Kwang Hwa Jung, Ji-Su Kim, Yun-Yong Park, Song Cheol Kim and Seokho Kim

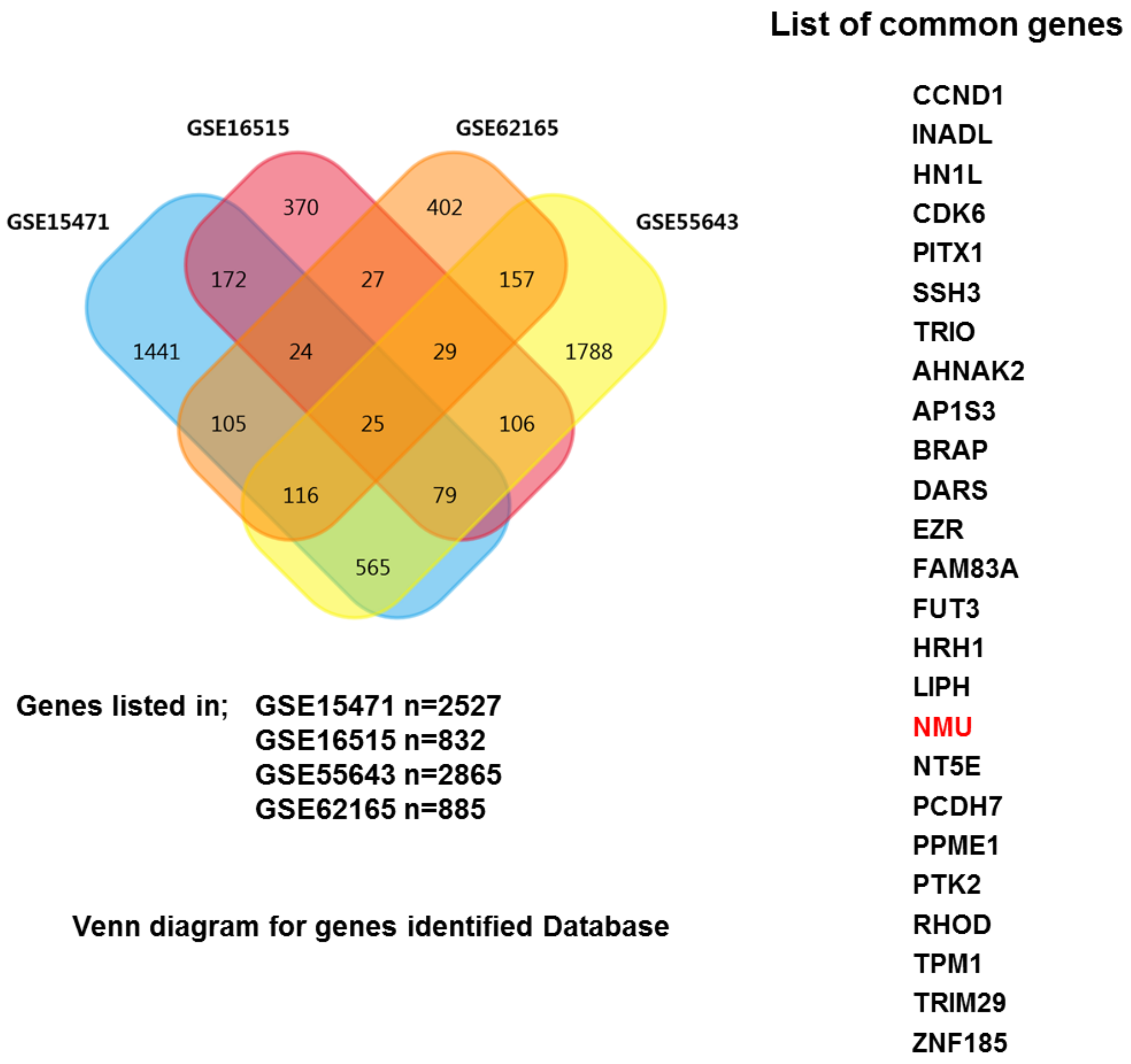

Figure S1. Venn diagram of differentially expressed genes of the GSE15471, GSE16515, GSE55643, and GSE62165 datasets for the comparisons in Figure 1A. The comparison identified 25 differentially expressed genes.

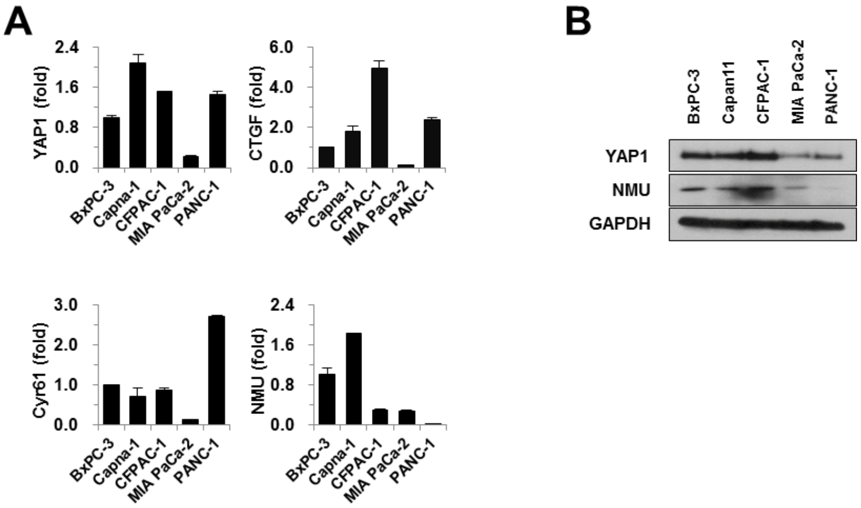

Figure S2. Expression levels of YAP1, its target genes, and NMU in human PDAC cells. (**A**), Real-time qPCR analysis of the gene expression of YAP1, CTGF, Cyr61, and NMU in BxPC-3, Capan-1, CFPAC-1, MIA PaCa-2, and PANC-1 cells. (**B**), Western blot analysis of YAP1 and NMU protein expression in PDAC cell lines.

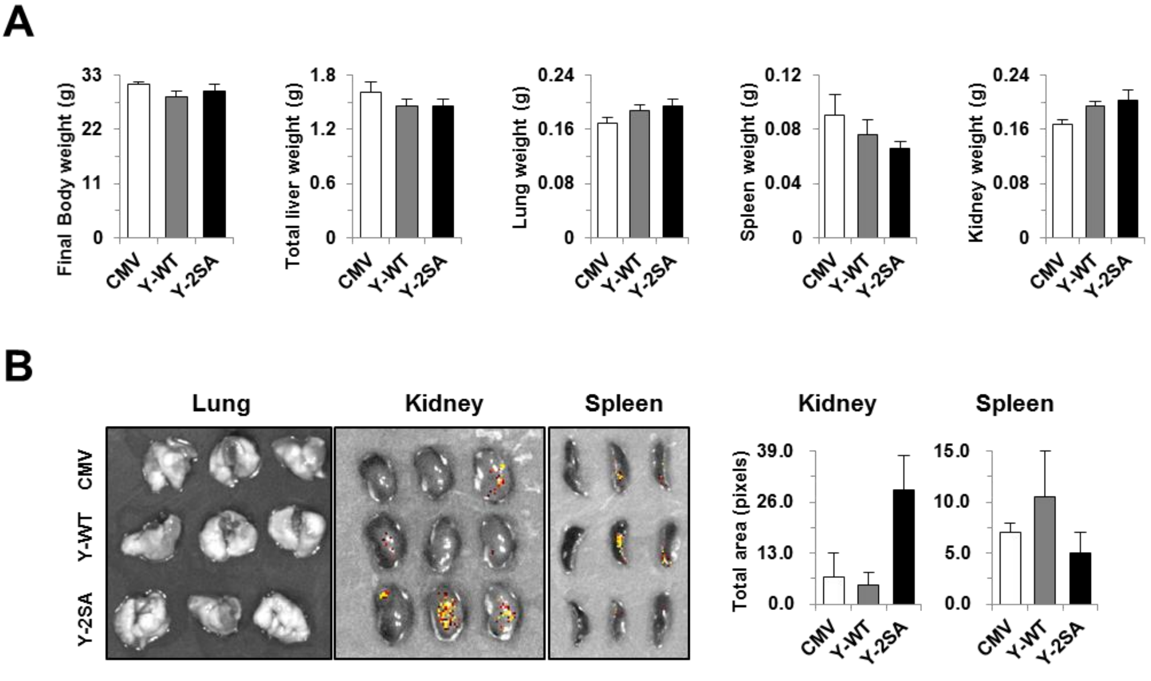

Figure S3. (**A**), Final body, liver, kidney, and spleen weight of CMV, Y-WT, and Y-2SA orthotopic tumor-bearing mice. (**B**), Bioluminescence images of the lung, kidney, and spleen and quantifications.

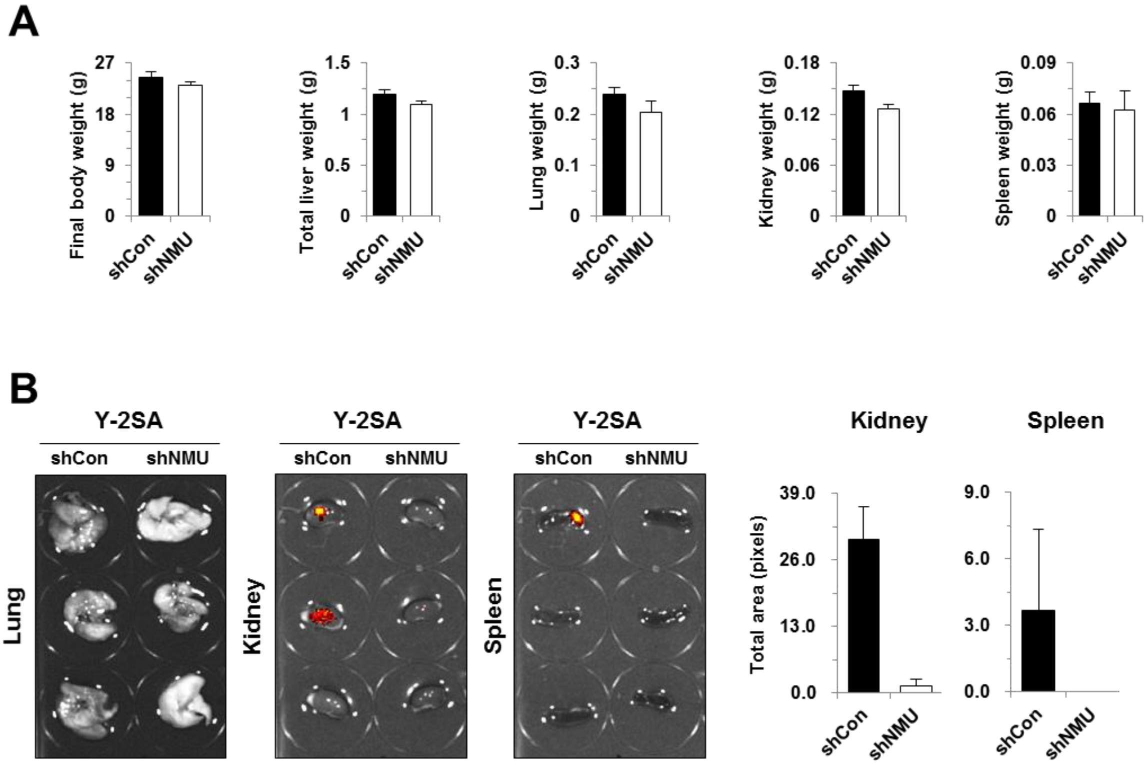

Figure S4. (**A**), Final body, liver, kidney, and spleen weight of Y-2SA-shCON and Y-2SA-shNMU orthotopic tumor-bearing mice. (**B**), Bioluminescence images of the lung, kidney, and spleen and quantifications.

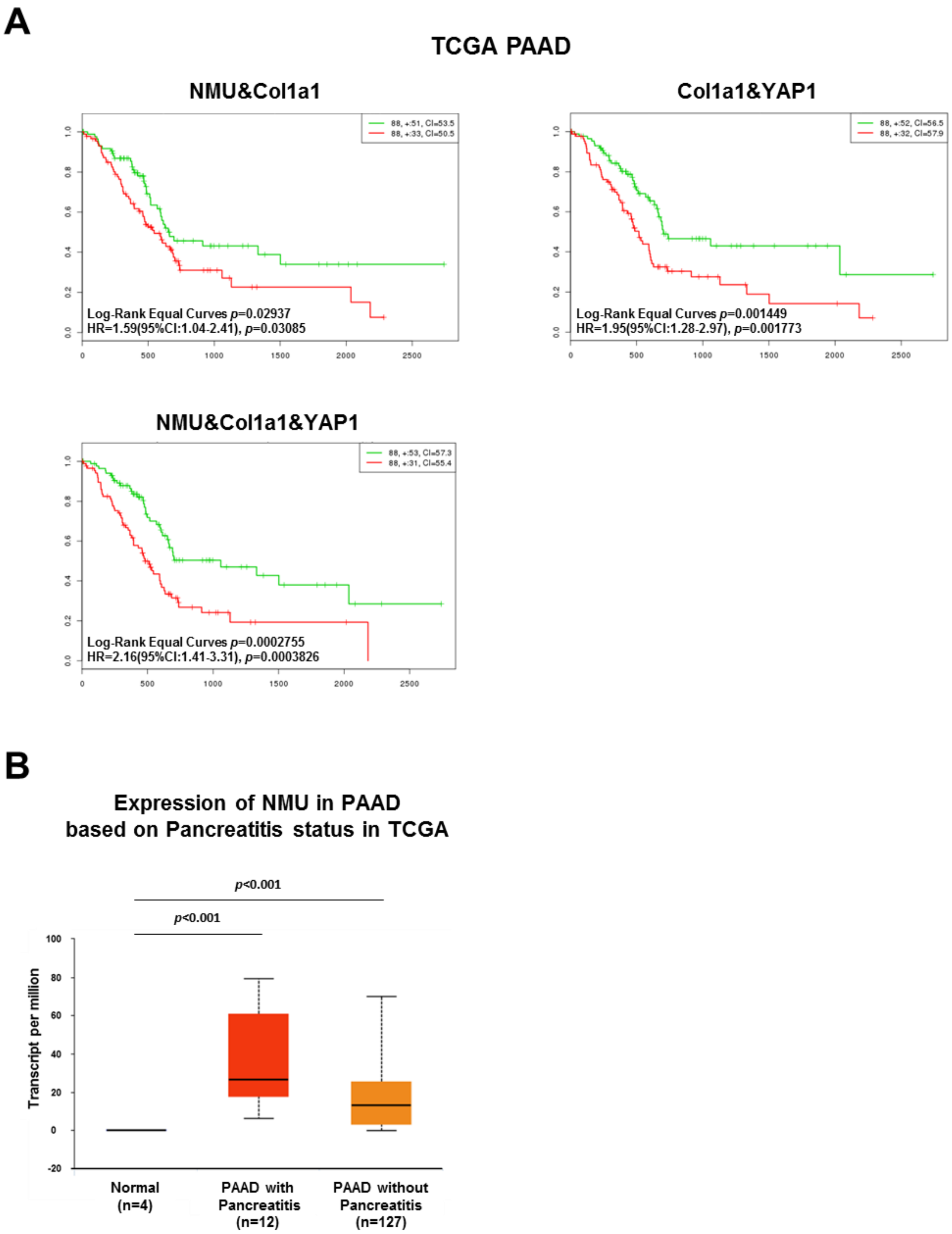

Figure S5. (**A**), Kaplan-Meier survival analysis of pancreatic adenocarcinoma patients from TCGA according to YAP1, NMU, and Col1a1 expression. *p*-values derived from the log-rank test are indicated in each comparison. (**B**), Kaplan-Meier survival analysis of patients with or without pancreatitis according to NMU expression. Values are expressed as the mean ± SEM.

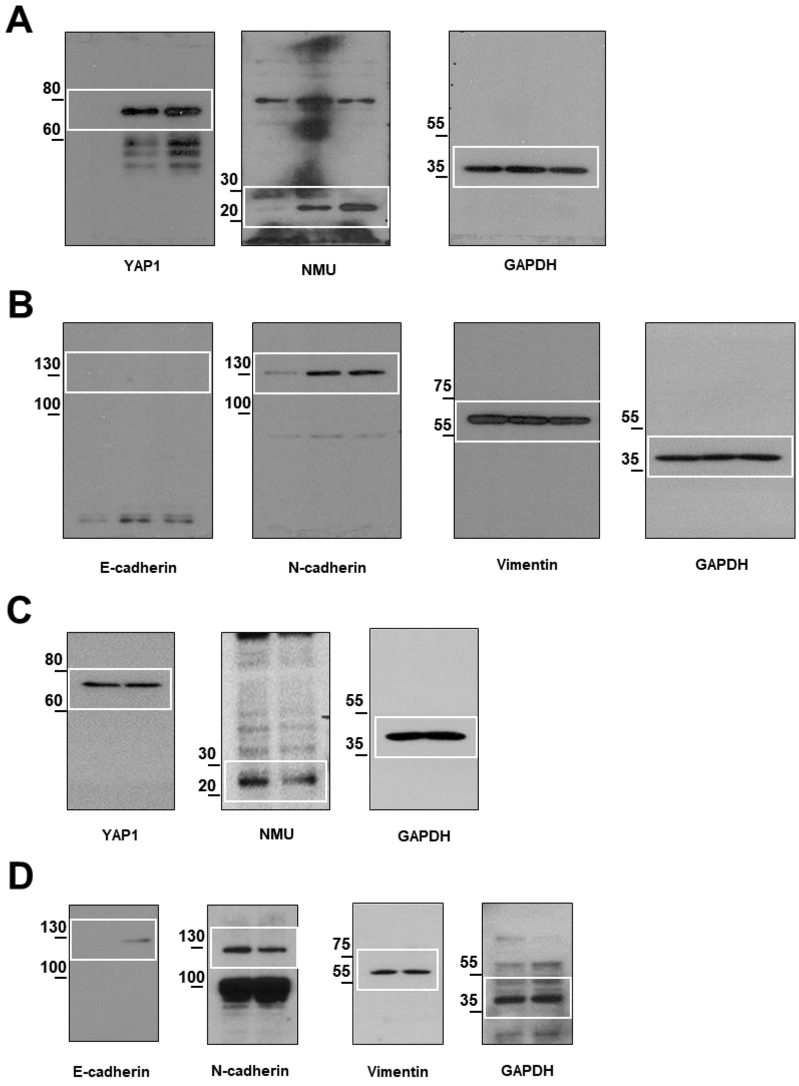

Figure S6. Original western blots. Western blots from Figure 3A (**A**), from Figure 3F (**B**), from Figure 4A (**C**), and from Figure 4H (**D**).

Table S1. Primer information.

Gene	Forward (5′–3′)	Reverse (5′–3′)
YAP1	ATGAGATGGATACAGGTGATA	TAAGGATGTCAGAACTCAAAG
NMU	TACTTGTTCGTCTTTTCTGTC	CTTCTGTGTCTTCGAATAATG
CTGF	GACATACCGAGCTAAATTCT	TCCGTACATCTTCCTGTAGTA
Cyr61	CCTGTGAATATAACTCCAGAA	TTGATACTATCCTCGTCACAG
E-cadherin	CGAGAGCTACACGTTCACGG	GTGTCGAGGGAAAAATAGGCTG
N-cadherin	GACGGTTCGCCATCCAGAC	TCGATTGGTTTGACCACGG
Vimentin	CAACCTGGCCGAGGACAT	ACGCATTGTCAACATCCTGTCT
GAPDH	CCACTCCTCCACCTTTGAC	ACCCTGTTGCTGTAGCCA

© 2019 by the authors. Licensee MDPI, Basel, Switzerland. This article is an open access article distributed under the terms and conditions of the Creative Commons Attribution (CC BY) license (http://creativecommons.org/licenses/by/4.0/).
